# Designing Nonconventional Luminescent Materials with Efficient Emission in Dilute Solutions via Modulation of Dynamic Hydrogen Bonds

**DOI:** 10.3390/molecules28135240

**Published:** 2023-07-06

**Authors:** Xuansi Tang, Bingli Jiang, Yongyang Gong, Yuxin Jin, Jiao He, Huihong Xie, Song Guo, Yuanli Liu

**Affiliations:** 1 Key Laboratory of New Processing Technology for Nonferrous Metal & Materials, Ministry of Education, Guilin University of Technology, Guilin 541004, China; tangxuansi@foxmail.com (X.T.); he19991205@163.com (J.H.); huihong0917@foxmail.com (H.X.); bobingjin@glut.edu.cn (S.G.); 2 College of Pharmacy, Guilin Medical University, Guilin 541199, China; jiangbingli@foxmail.com (B.J.); jyx1140257079@163.com (Y.J.)

**Keywords:** nonconventional luminescent polymer, clustering-triggered emission, dynamic hydrogen bonds, Fe^3+^ detection, cell imaging

## Abstract

Nonconventional luminescent materials (NLMs) which do not contain traditional aromatic chromophores are of great interest due to their unique chemical structures, optical properties, and their potential applications in various areas, such as cellular imaging and chemical sensing. However, most reported NLMs show weak or no emission in dilute solutions, which severely limits their applications. In this work, dynamic hydrogen bonds were utilized to design NLMs with efficient emission in dilute solutions. To further validate the results, polymers P1 and P2 were successfully prepared and investigated. It was found that the luminescence quantum efficiency of P1 and P2 at a concentration of 0.1 mg/mL in water solution was 8.9 and 0.6%, respectively. The high efficiency can be attributed to the fact that polymer P1 has more intra- or intermolecular dynamic hydrogen bonds and other short interactions than P2 in dilute solutions, allowing P1 to achieve the through-space conjugation effect to increase the degree of system conjugation, restrict molecular motion, and decrease nonradiative transitions, which can effectively improve luminescence. In addition, polymer P2 exhibits the characteristics of clustering-triggered emission, excitation wavelength-dependent and concentration-dependent fluorescence properties, excellent photobleaching resistance, low cytotoxicity, and selective recognition of Fe^3+^. The present study investigates the manipulation of luminescence properties of NLMs in dilute solutions through the modulation of dynamic hydrogen bonds. This approach can serve as a semi-empirical technique for designing and building innovative NLMs in the times ahead.

## 1. Introduction

High-efficiency organic luminescent materials have been widely used in many fields, such as electronic devices [1,2,3], chemical sensing [4,5,6], phototherapy [7,8,9], and bioimaging [10,11,12,13]. However, these conventional, efficient, organic luminescent materials are usually prepared by C-C coupling, C-N coupling, or other reactions between aromatic units, which require not only expensive noble metal catalysts but also harsh synthesis conditions [14]. In addition to the high biotoxicity of aromatic compounds, fused ring aromatic compounds may endanger human health and pollute the environment [15]. In recent years, organic luminescent materials without aromatic units with good biosafety that exhibit bright emission in the aggregated state or at high concentrations known as clustering-triggered emission (CTE) have attracted increasing attention [16,17,18,19,20,21,22], and reported materials with CTE characteristics include cellulose [23,24], poly(amido) poly(amidoamine) (PAMAM) dendrimers [25], hyperbranched and linear polyethyleneimines [26], hyperbranched poly(amido), polyacrylonitrile [27], poly-L-aspartic acid [28], nonaromatic amino acids [19], hyperbranched polysiloxanes [29,30,31], and cyanoacetic acid [32], which are rich in hydroxyl, amino, carboxyl, carbonyl, and heteroatoms in their chemical structures. Regarding the CTE mechanism, it is assumed that the intramolecular or intermolecular short interactions of nonconventional luminophores at high concentrations or in solid states make the electron cloud more evenly distributed in the whole system such that it creates a space conjugation effect, which expands the degree of conjugation of the system and realizes the CTE phenomenon [17].

However, many reported nonconventional luminescent materials (NLMs) show bright luminescence only at high concentrations or in solid states, and high concentrations will make cells lose water and die in cell imaging, limiting their further applications. Therefore, there is an urgent need to develop novel NLM compounds with high photoluminescence efficiency (*Φ*) in dilute solutions. Up to now, only a few papers have sporadically reported the phenomenon of effective luminescence of NLMs in dilute solutions, and these studies have relied on quantum chemical principles based on static isolated systems, which do not truly reflect the real state of the solution system. For example, Gong et al. reported that a random copolymer, Poly-L-aspartic acid, containing a rigid hydrophobic segment and a flexible hydrophilic segment can achieve a luminescence efficiency of 4.6% by self-assembling into nanoclusters in dilute solution [28]. By modifying cyclodextrin (CD) with amino acids, Mao et al. created unique clusteroluminogens that allow for intermolecular and intramolecular clustering of chromophores within the confined space of CD and efficient generation of fluorescence, ranging from blue to cyan, even in dilute solutions [33]. Tang et al. applied quantum chemical principles to construct two new compounds, LJ-1 and LJ-2, which possess both electron donor and acceptor groups, by correctly considering π→π* transitions and intramolecular hydrogen bonding. Both compounds emit bright green fluorescence in dilute solutions with a high quantum yield of up to 59.8% and exhibit targeting to lysosomes and pH sensitivity [34]. In addition, quantum chemistry can only be used to study isolated systems of one or two hundred atoms, and it is not suitable for studying all atoms in polymeric systems.

According to the CTE mechanism, strengthening the intramolecular through-space conjugation effect by intramolecular or intermolecular interactions can achieve significant fluorescence or phosphorescence emission of NLMs at high concentrations or in solid states. Therefore, there are two ways to improve the luminescence efficiency of NLMs in solution states. The first is to enhance the intramolecular or intermolecular interactions, and this method can achieve a luminescence efficiency of 59.8% for NLMs in dilute solutions (2 × 10^−5^ mol/L) [34]. However, enhancing the strength of hydrogen bonds in dilute solutions is difficult, and in addition to choosing reasonable functional groups for NLMs, the interactions between the functional groups of NLMs in solution should be considered. The second approach is to increase the number of hydrogen bonds and other short interactions (≤3.5 Å). The hydrogen bond strength in the first approach can be calculated using quantum chemical methods, while the number of short interactions and hydrogen bonds in the second approach can be obtained quickly using molecular dynamics (MD) calculations.

**Figure 1:**
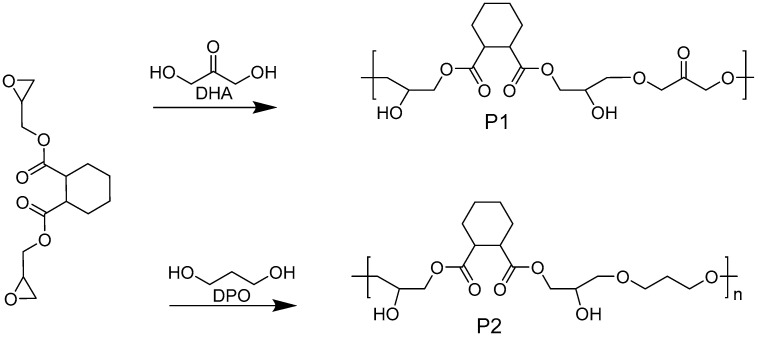
Synthesis pathways and chemical structures of target polymers P1 and P2.

In order to gain insight into the experimental phenomenon of enhanced luminescence intensity for NLMs with increasing concentration in solution and to verify whether dynamic hydrogen bond engineering can be used to construct NLMs with efficient properties in dilute solutions, diglycidyl 1,2-cyclohexanedicarboxylate (DCD) was used to synthesize polymers P1 and P2 by ring-opening reactions with 1,3-dihydroxyacetone (DHA) and 1,3-Propanediol (PDO), respectively (Figure 1). The effects of concentration and the number of hydrogen bond donors/acceptors and short interactions on their optical properties in dilute solutions were investigated by experiments and MD simulations. The results showed that the fluorescence emission/excitation wavelengths of P1 and P2 were 480/400 nm and 420/350 nm, respectively, and the fluorescence quantum yield of the P1 polymer reached 8.9% in a 0.1 mg/mL concentration solution, while P2 was essentially nonemissive at the same concentration. MD simulations show that P1 has more intra- or intermolecular hydrogen bond donors/acceptors and short interactions than P2 for the same number of polymer repeating units; furthermore, increasing the concentration can enhance the number of hydrogen bonds donors/acceptors and short interactions in the system. This work demonstrates that dynamic hydrogen bond engineering can be used as a semi-empirical method to design increasingly novel NLMs that emit efficiently in dilute solutions.

## 2. Results

### 2.1. Molecular Dynamics Simulation

In terms of chemical structure, polymer P1 has more typical hydrogen bond acceptors (carbonyl groups) compared with polymer P2. The variation in the quantity of hydrogen bond donors/acceptors results in a marked difference in the number of hydrogen bonds and short-range interactions. MD simulations in water solution at 298.15 K using the Gromacs package [35] with the general AMBER force field (GAFF) [36] generated by Sobtop [37] were performed to elucidate the dynamic hydrogen bonds of both P1 and P2 in solution. It is worth noting that, when analyzing the simulation results, only hydrogen bonds with bond angles less than or equal to 30 degrees and atomic distances between hydrogen bond donors and acceptors less than or equal to 3.5 angstroms should be considered.

**Figure 2:**
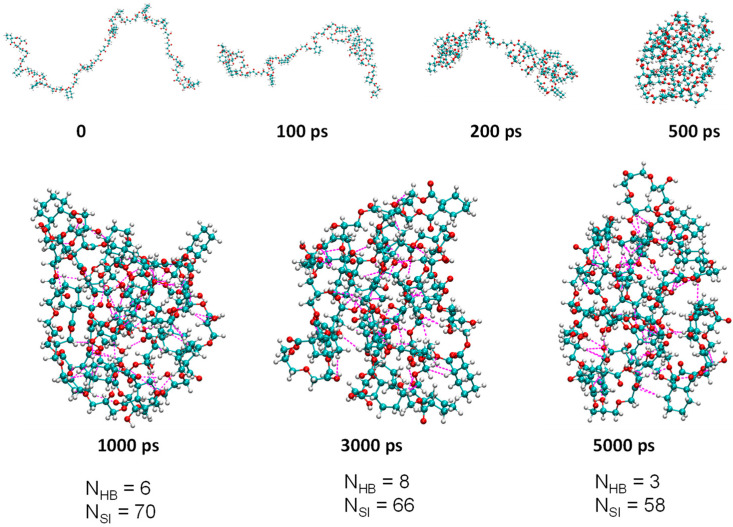
The aggregation state of P1 in the 10 nm × 10 nm × 10 nm cubic water box at different times and the number of hydrogen bonds (N_HB_) and short interactions (N_SI_) of the molecules at different times. The magenta dashed lines represent partial hydrogen bonds and short interactions.

The model polymers were constructed with 11 repeat units for both P1 and P2. MD simulations were performed in a 10 nm × 10 nm × 10 nm cubic water box with a 5 ns simulation time. As can be seen from Figure 1 and Figure S1, chain-like P1 and P2 formed nanoaggregates in aqueous solution with increasing time, which were stabilized in the aggregated state after 500 ps. Furthermore, the number of hydrogen bonds (N_HB_) and short interactions (N_SI_) within P1 in aqueous solution varied dynamically at different times (Figure 2). Therefore, it is not sufficient to study NML materials solely in terms of the type and strength of hydrogen bonds; it is also necessary to examine the dynamics of hydrogen bonds (including short interactions). It is worth noting that as the polymer content (amount) increases, the polymer is able to form large aggregates in the solution, as shown in Figure S2, which is consistent with many experimental phenomena reported in the literature, where the particle size of NLMs increases with increasing concentration.

**Figure 3:**
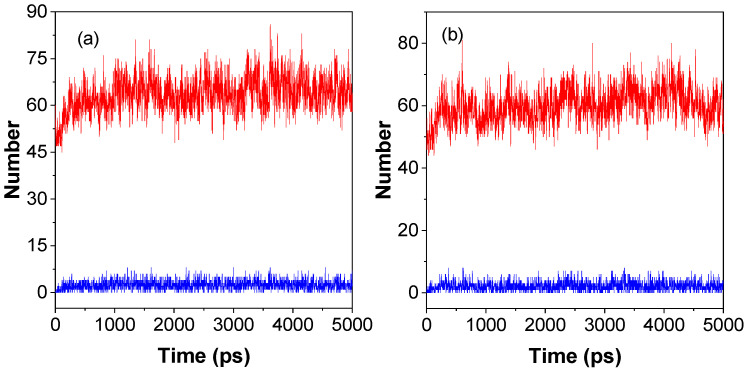
Molecular dynamics simulations of intramolecular dynamics of hydrogen bonding and short interactions for P1 (**a**) and P2 (**b**) in a 10 nm × 10 nm × 10 nm cubic water box. The red and blue lines indicate the numbers of hydrogen bonds and short interactions, respectively.

Lifetime (τ_HB_) is one of the most important parameters of dynamic hydrogen bonds; it is the time from initial formation to complete rupture of a hydrogen bond under specific conditions and can be attributed to the formation and rupture of hydrogen bonds. It can be calculated by averaging the autocorrelation functions (either 0 or 1) of all H-bonds according to previous studies in the literature [38]. The τ_HB_ values for P1 and P2 in water solution were 1.5 and 1.6 ps, respectively; the almost identical values of the lifetimes indicate that they are influenced by the type of hydrogen bond donors and hydrogen bond acceptors. The number of donors/acceptors of hydrogen bonds is closely related to the chemical structure of the polymer, and these numbers were 24/100, and 24/89 for P1 and P2, respectively. The P1 polymer repeat unit has a typical hydrogen bond acceptor (C = O) compared with the P2 polymer repeat unit. Thus, P1 has more hydrogen bond acceptors than P2. As shown in Figure 3, the number of hydrogen bonds and the number of short interactions increased with time from 0 to 500 ps due to the gradual aggregation of the polymer, from chains to nanoclusters (Figure 2). Then, the numbers of hydrogen bonds and short interactions changed dynamically with time. The average number of intramolecular hydrogen bonds/short interactions (N_HB_/N_SI_) within a molecule from 1000 ps to 5000 ps for P1 and P2 was 3/65 and 2/61, respectively. These results indicate that P1 can form more hydrogen bonds and short interactions than P2 in the solution state, which suggests that P1 is more conducive to restricting molecular motion, reducing nonradiative transitions, and enhancing luminescence compared with P2. Moreover, according to the mechanism of cluster-trigged emission, more hydrogen bonds and short interactions can enhance the through-space conjugation effect and enable NLMs to luminesce effectively in solution.

### 2.2. Synthesis

MD simulations showed that P1 had a higher number of intra-/intermolecular hydrogen bonds and shorter interactions compared with P2 in a 1000 nm^3^ water box. To examine the effects of dynamic hydrogen bonds on the optical properties of NLMs, polymers P1 and P2 were successfully synthesized, and their optical properties were studied. First, P1 and P2 were characterized by Fourier transform infrared (FTIR) spectroscopy, gel permeation chromatography (GPC), and nuclear magnetic resonance (NMR). As shown in the FTIR spectra (Figures S2–S5), the main absorbance bands around 3403 cm^−1^ and 2940 cm^−1^ can be assigned to asymmetrical OH stretching vibrations and C-H stretching vibrations, respectively. In addition, the characteristic peak at 1731 cm^−1^ corresponding to the stretching vibration of -C = O and the characteristic peak at 1126 cm^−1^ corresponding to the stretching vibration of C-O-C can be easily observed. The weight average molecular weight (Mw)/number averaged molecular weight (Mn) for P1 and P2 was 3755/1715 and 1984/1145 daltons, respectively (Table S1). The polydispersity index (PDI) was 2.2 and 1.7, respectively. The above results indicate that the molecular weight broadness is in the normal range.

### 2.3. Optical Properties of P1 and P2 in Solution

**Figure 4:**
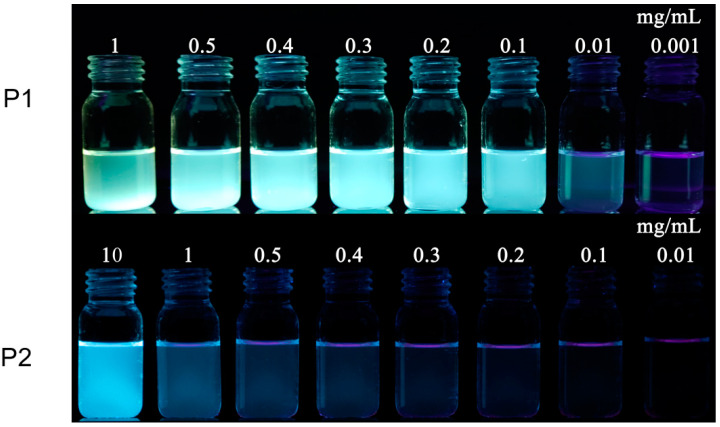
Photographs of ethanol solutions of P1 and P2 at different concentrations under 365 nm UV light.

According to many previous reports, NLMs without aromatic conjugated units can only emit light in highly concentrated solutions, while they hardly emit in dilute solutions. As shown in Figure 4, it was quite unexpected to observe bright blue-green fluorescence emission in dilute solutions (0.1 mg/mL) for P1 under the irradiation of a 365 nm UV lamp. However, the P2 solution emitted essentially no fluorescence at a concentration of ≤1.0 mg/mL. Subtle differences in chemical structure exhibit completely different optical properties, so it is worthwhile to investigate the photophysical properties of both P1 and P2 in depth.

**Figure 5:**
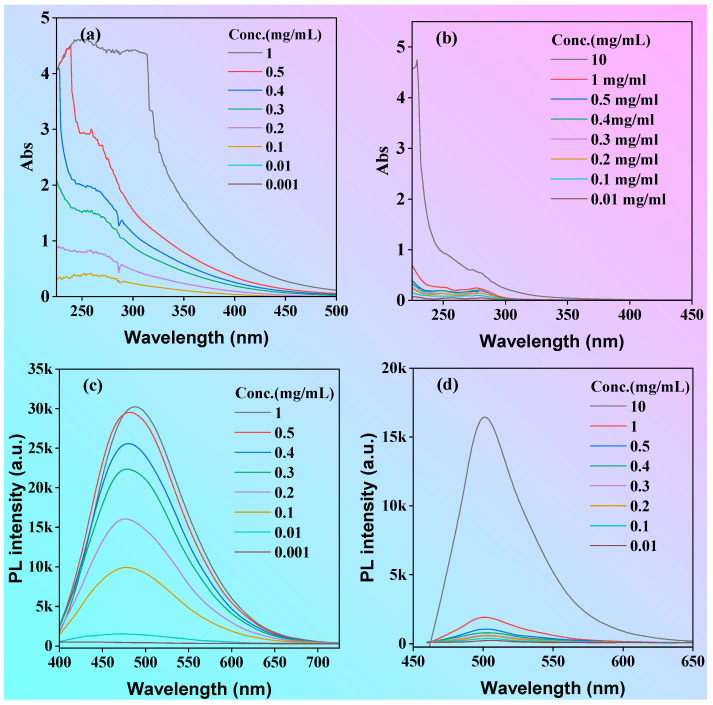
(**a**) Absorption (**a**,**b**) and fluorescence emission (**c**,**d**) spectra of P1 (**a**,**c**) and P2 (**b**,**d**) in ethanol solution. λex = 375 nm.

To acquire more insights into the photophysical properties, the absorption and emission spectra of both P1 and P2 in ethanol solution at different concentrations were investigated, as shown in Figure 5. The absorbance intensity of the P1 solution increased significantly with the increase in concentration (Figure 5a,b), whereas the absorbance intensity of polymer P2 changed little with the increase in concentration at <1 mg/mL but increased significantly when the concentration went up to 10 mg/mL. Due to the polydispersity of the molecular weight of the polymers, we used mass extinction absorption (L·g^−1^·cm^−1^) to investigate the magnitude of their light absorption capacities. The mass extinction coefficients for the P1 and P2 solutions at a concentration of 1 mg/mL were 4.41 and 0.05 L·g^−1^·cm^−1^ at the peak of 300 nm, respectively. The mass extinction coefficient of P1 was much greater than that of P2. According to the semi-empirical Equation (1), the (molar) extinction absorption of the solution is proportional to the luminescence efficiency. Therefore, it can be tentatively concluded that P1 has a higher luminescence efficiency than P2, which is consistent with the phenomena shown in the luminescence photographs of the P1 and P2 solutions under the UV light (Figure 2).

(1) $$\phi_{f}=10^{4}\varepsilon_{max}$$

where *Φ_f_* and *ε_max_* are the photoluminescence efficiency and maximum molar extinction coefficient, respectively.

In order to qualitatively study the luminescence in solution, the emission spectra of P1 and P2 in solution were examined. As shown in Figure 5c, the fluorescence emission curve appeared to be parallel to the x-axis at concentrations below 0.1 mg/mL, whereas the fluorescence intensity of the P1 solution increased significantly when the concentration was above 0.1 mg/mL. Different from P1, the luminescence intensity of P2 was very weak at concentrations less than or equal to 1 mg/mL, and the fluorescence intensity significantly increased until concentrations were greater than 10 mg/mL (Figure 5d). The correlation between fluorescence intensity and concentration was comparable to that observed with the absorption spectra. The fluorescent quantum yields of P1 and P2 for the 0.1 mg/mL concentration solutions were 8.9 and 0.6%, respectively. According to the results of molecular dynamics studies, compared with P2, the more effective luminescence of P1 in dilute solutions can be attributed to more hydrogen bonds and other short interactions in solution. These interactions can have two effects: firstly, they can greatly restrict molecular motion, reducing nonradiative transitions and enhancing luminescence; secondly, they can expand the degree of conjugation, triggering visible light emission through through-space conjugation. This result indicates that P1 is a promising candidate for application in cellular imaging.

**Figure 6:**
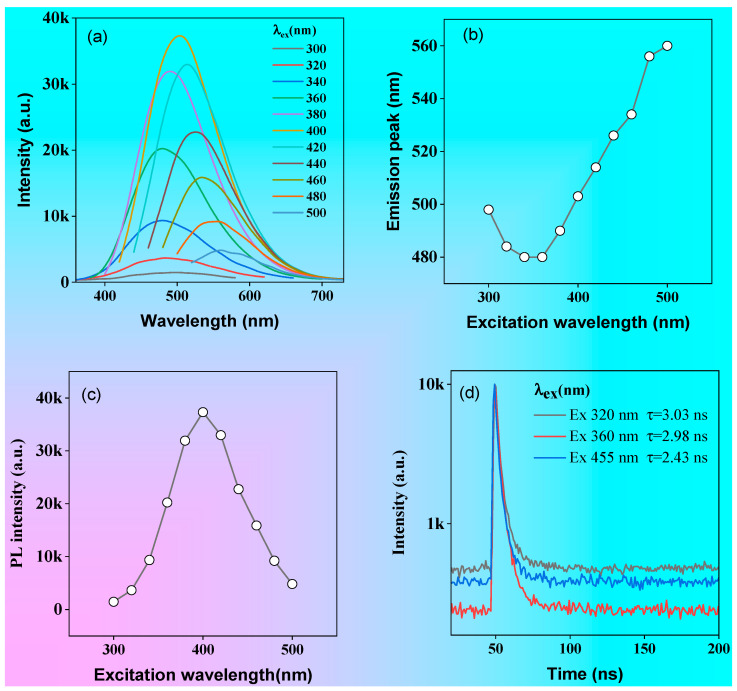
Emission spectra of the P1 solution (1 mg/mL) at different excitation wavelengths (**a**); the change in emission peaks with different excitation wavelengths (**b**); the change in intensity for the maximum emission peak with different excitation wavelengths (**c**); the fluorescence lifetime at 400 and 435 nm tested with 320, 360, and 455 nm picosecond lasers (**d**).

The emission spectra of the P1 solution under different excitation wavelengths are shown in Figure 4a. As the excitation wavelength changed from 300 to 340 nm, the fluorescence emission peak showed blue shift from 520 to 509 nm, but when the excitation wavelength was greater than 360 nm the fluorescence emission peak gradually exhibited red shift as the excitation wavelength increased (Figure 6b). In addition, the fluorescence emission intensity exhibited excitation-wavelength-dependent properties (Figure 6c).

The fluorescence intensity increased with the increase in the excitation wavelength when the excitation wavelength was less than 400 nm but gradually decreased with the increase in the excitation wavelength when the excitation wavelength was greater than 400 nm (Figure 6c). Furthermore, the fluorescence lifetimes of P1 varied obviously at different excitation wavelengths (Figure 6d), these being 3.03, 2.98, and 2.43 ns for 320, 360, and 455 nm picosecond laser excitations, respectively. The diversity of fluorescence intensities, emission peaks, and fluorescence lifetimes for polymer P1 at different excitation wavelengths indicated the excitation-wavelength-dependence of their optical properties, which can be attributed to the formation of different excitons in solution.

**Figure 7:**
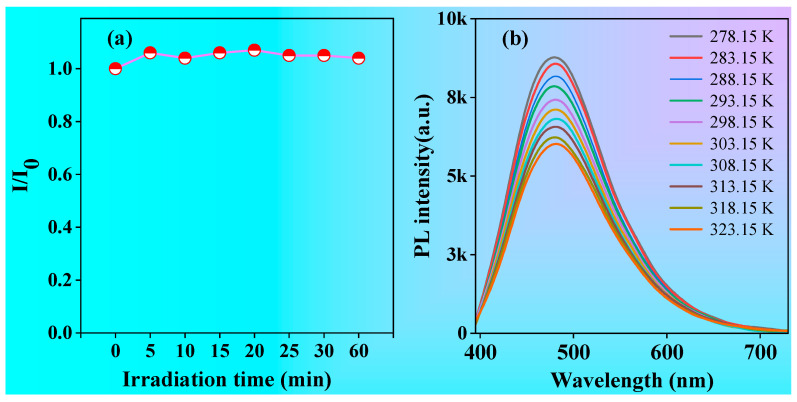
(**a**) The changes in emission peaks for the P1 solution upon white light irradiation at different times. (**b**) Fluorescence emission spectra of the P1 solution with different temperatures. Concentration: 1 mg/mL; λ_ex_ = 375 nm.

As we all know, photostability or resistance to photobleaching is an important parameter for organic luminescent materials, especially in the application of cellular laser confocal imaging. In order to study the photostability of polymer P1, a diluted ethanol–water solution of P1 at 1.0 mg/mL was continuously irradiated with a UV lamp at 365 nm while its fluorescence intensity was recorded. As shown in Figure 7a, the fluorescence intensity of P1 showed little change when continuously irradiated under 365 nm UV light, suggesting its good photostability, which will be beneficial for its potential applications, such as cell imaging. In addition, temperature has a significant influence on the optical properties of organic luminescent materials, as it can affect intramolecular and intermolecular interactions. To investigate the effect of temperature on the optical properties of polymer P1, the emission spectra of P1 in solutions were investigated from 278.15 to 323.15 K. As shown in Figure 7b, the intensity of the emission peak of P1 gradually decreased with the increase in temperature, which was primarily due to the fact that as the temperature rose, the viscosity of the system reduced, and then the thermal motion of the molecules increased, thus leading to the enhanced rate of nonradiative transitions and the reduced luminescence intensity. At low temperature, the intermolecular movement of the polymer is difficult and restricted, which leads to reduction in the rate of nonradiative transition and increase in the intensity of luminescence. The linear decrease in fluorescence intensity with increasing temperature means that **P1** can be used for temperature sensing [39].

### 2.4. Polymer P1 for Iron Ion Detection

**Figure 8:**
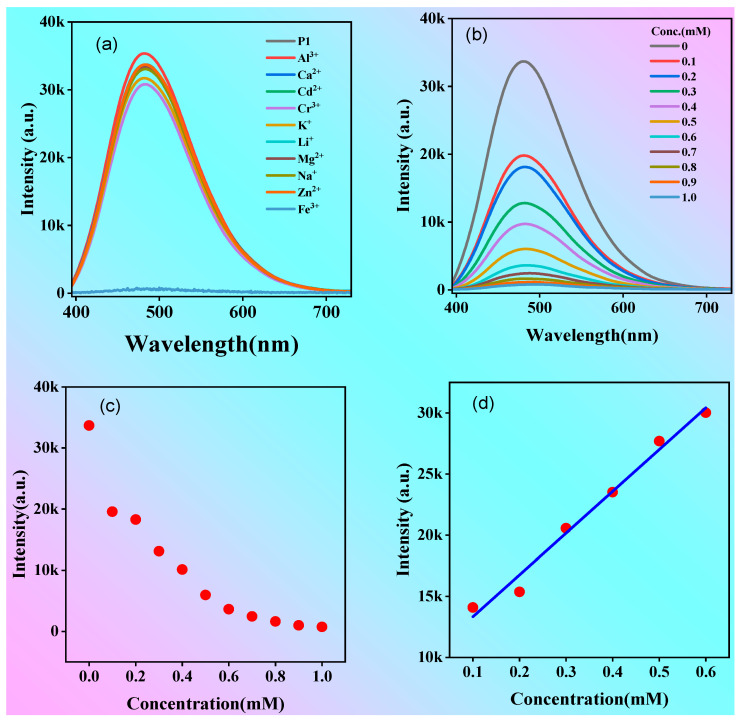
(**a**) Fluorescence spectra of P1 solution after adding different metal ions (concentration: 1 mM). (**b**) Fluorescence spectra of P1 solution after adding different concentrations of Fe^3+^. (**c**) Relationship between fluorescence intensity and concentration of P1 solution. (**d**) The change in fluorescence intensity ΔI = I_0_ − I under different Fe^3+^ concentrations. Concentration of P1 solution: 1 mg/mL; λ_ex_ = 375 nm.

Due to the presence of numerous hydroxyl and carboxyl groups, P1 exhibits efficient emission in dilute solutions, allowing it to form complexes with metal ions and exhibit a distinctive ionic recognition effect. To test whether the polymer could be used to detect metal ions, the fluorescence of the P1 solution (1 mg/mL) containing 10 metal ions was measured, as shown in Figure 8a. Impressively, the fluorescence intensity was almost parallel to the x-axis when Fe^3+^ was added to the solution; however, the addition of other metal ions to the P1 solution had almost no effect on the fluorescence intensity. The above results suggest the excellent selective quenching effect of iron ions (Fe^3+^) for P1.

Fe^3+^ is one of the essential trace elements for human growth and development. At the same time, it is closely related to a range of diseases, such as diabetes, liver and kidney damage, and anemia [40,41,42,43,44]. In other words, the detection of Fe^3+^ is important for the early identification and diagnosis of diseases caused by abnormal Fe^3+^ concentrations in the human body. The detection limit of P1 for Fe^3+^ was determined. When different concentrations of Fe^3+^ solutions were added to the P1 solution, the maximum emission peak was always at 480 nm when the excitation wavelength was 375 nm, but the intensity of the emission peak decreased continuously (Figure 8b,c). The limit of detection (LOD) was 33.67 μM according to the reported 3S/K calculation method [45] (S in Figure 8d is the standard deviation from blank and K is the slope of the linear curve). The results indicate that polymer P1 has good sensitivity to Fe^3+^ and thus possesses great potential as a probe for Fe^3+^.

### 2.5. Cell Imaging Studies

**Figure 9:**
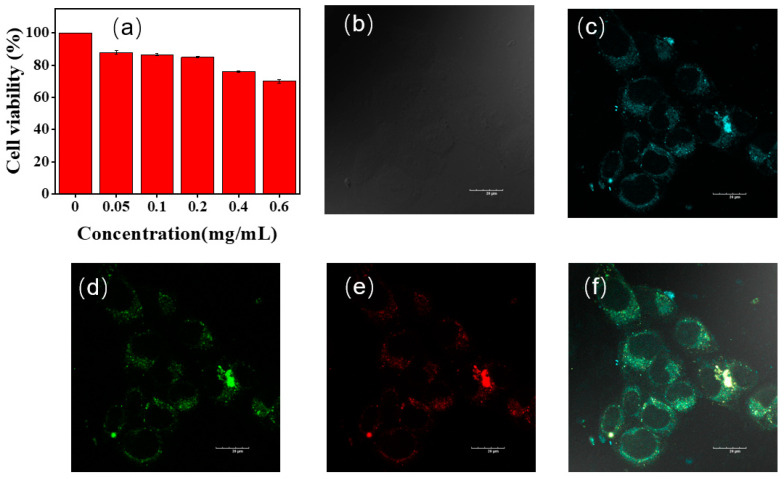
(**a**) Cell viability of HeLa cells incubated with different concentrations of P1 for 24 h. Confocal microscopy images of HeLa cells incubated with P1 (**b**–**f**). (**c**–**f**) are bright field, blue, green, red, and merged channel images, respectively. Scale: 20 μm; concentration: 0.5 mg/mL.

NLMs without aromatic groups which exhibit CTE properties are often used for cell imaging due to their low toxicity [46,47,48,49]. To validate the potential of P1 for cell imaging and labelling applications, we used standard methylthiazolyl diphenyltetrazolium bromide (MTT) assays to evaluate the cytotoxicity of P1 at different concentrations on HeLa cells. As shown in Figure 9a, cell viability exceeded 85% when the concentration of P1 was less than 0.2 mg/mL. The cell viability decreased to 70% when the concentration of polymer P1 reached 0.6 mg/mL, suggesting low cytotoxicity and great potential of P1 for biological imaging applications.

Then, HeLa cells incubated with polymer P1 (0.5 mg/mL) for 1 h were analyzed by confocal laser microscopy (Figure 9b–f). Due to the excitation-wavelength-dependence of P1, the emissions from the blue, green, and red channels were collected in three different channels to obtain cell images. As can be seen from the bright field in Figure 7b, the morphology of all HeLa cells remained normal. The cytoplasm of the HeLa cells showed blue, green, and red fluorescence emission when excited at 405, 488, and 561 nm, respectively. The results indicate that polymer P1 is capable of bioimaging at the cellular level with multicolor imaging.

## 3. Conclusions

In summary, the dynamic hydrogen bonds and other short interactions in solutions of the model polymers P1 and P2 with the same number of repeating units were first investigated by means of MD. It was found that polymer P1 has more intra- or intermolecular hydrogen bonds and other short interactions in solution than P2 and that these can be effectively modulated by controlling the number of hydrogen bond acceptors of the polymer monomers. To further validate the results of the MD study, polymers P1 and P2 were prepared, and their photophysical properties were investigated. It was found that the mass extinction values at 300 nm for 1 mg/mL solutions of P1 and P2 were 4.45 and 0.05 L/mol/cm, respectively, and that the luminescence quantum efficiencies for 0.1 mg/mL solutions of P1 and P2 were 8.9 and 0.6%, respectively. The excellent photophysical properties of P1 can be attributed to its rich intra- or intermolecular hydrogen bonds and other short interactions in dilute solutions, which allows it to increase the degree of system conjugation, restrict molecular motion, and decrease nonradiative transitions, thereby leading to effective luminescence. In addition, P1 exhibits clustering-induced luminescence, excitation-wavelength-dependent and concentration-dependent fluorescence properties, excellent photobleaching resistance, and selective recognition of Fe^3+^. Although the relationship between dynamic hydrogen bonds and optical properties has yet to be investigated at a large-scale in depth, this work is a preliminary study of the luminescence properties of NLMs in dilute solutions conducted using molecular dynamics to study dynamic hydrogen bonds, which can be used as a semi-empirical method to design and construct increasingly novel NLMs.

## Data Availability

Data is contained within the article.

## Supplementary Materials

The following supporting information can be downloaded at: https://www.mdpi.com/article/10.3390/molecules28135240/s1, Figure S1. The aggregation state of P2 in 10 nm × 10 nm × 10 nm cubic water box at different times, and the number of hydrogen bonds (N_HB_) and short interactions (N_SI_) of the molecules at different times, the magenta dashed lines represent partial hydrogen bonds and short interactions. Figure S2. Molecular dynamics simulations of the aggregated state of a P1 polymer with polymer number 10 and repeating unit 11 in solution at different times. Figure S3. ^1^H NMR spectrum of P1 and the corresponding synthetic raw materials. Figure S4. FT-IR spectrum of P1 and the corresponding synthetic raw materials. Figure S5. ^1^H-NMR spectrum of P2 and the corresponding synthetic raw materials. Figure S6. FT-IR spectrum of P2 and the corresponding synthetic raw materials. Table S1. Molecular weight characterization of P1 and P2.
