# Supplementary material for: Designing Nonconventional Luminescent Materials with Efficient Emission in Dilute Solutions via Modulation of Dynamic Hydrogen Bonds
Source: Molecules. 2023 Jul 6;28(13):5240. doi: 10.3390/molecules28135240 (PMC10343347; doi:10.3390/molecules28135240)
Supplement: Supplementary file 1 [file molecules-28-05240-s001.zip › molecules-2428875-supplementary.pdf]

Supporting information

Designing Nonconventional Luminescent Materials with Efficient Emission in Dilute Solutions via Modulation of Dynamic Hydrogen Bonds

Xuansi Tang [1], Bingli Jiang [2], Yongyang Gong [1,*], Yuxin Jin [2], Jiao He [1], Huihong Xie [1], Song Guo [1,*] and Yuanli Liu [1,*]

[1] Key Laboratory of New Processing Technology for Nonferrous Metal & Materials, Ministry of Education, Guilin University of Technology, Guilin 541004, China; tangxuansi@foxmail.com (X.T.); he19991205@163.com (J.H.); huihong0917@foxmail.com (H.X.)

[2] College of Pharmacy, Guilin Medical University, Guilin 541199, China; jiangbingli@foxmail.com (B.J.); jyx1140257079@163.com (Y.J.)

* Correspondence: yygong@glut.edu.cn (Y.G.); bobingjin@glut.edu.cn (S.G.); lyuanli@glut.edu.cn (Y.L.)

Experimental section

Materials

1, 3-Dihydroxyacetone was purchased from Bide Pharmatech Ltd. Diglycidyl 1,2-cyclohexanedicarboxylate, tetrabutylammonium bromide, and absolute ethanol were purchased from Adamsa Reagents Co.Ltd. Tetrahydrofuran (THF) was purchased from Xilong Scientific Co.Ltd. Ultrapure water was prepared by ultrapure water preparation machine (AXLM1820). Fetal bovine serum (FBS), trypsin-EDTA digestion solution, penicillin streptomycin (100×) was purchased from Sollerbauer Technology Ltd. thiazolyl blue tetrazolium bromide (MTT) was obtained from Thermo Fisher Scientific Ltd and HeLa cells were purchased from the Cell Bank of the Chinese Academy of Sciences. All reagents and solvents are analytically pure grade.

Measurements

FT-IR spectra was carried out on a Fourier transform infrared spectrometer (Nicolet Nexus 470). ^{1}H NMR spectra was recorded on a Bruker AVANCE III HD 500 MHz spectrometer in C_2D_6SO.The M_W and the polydispersity index (PDI) of Z were measured using a Waters 1515 gel permeation chromatography (GPC) system which is calibrated with linear polystyrene calibration standards, THF was used as the mobile phase. The UV-vis absorption spectra were tested from UV spectrophotometer (Lambda 365). The fluorescence spectra were obtained with FluoroMax-4 fluorescence spectrometer and QuantaMater 8000 fluorescence spectrometer. The fluorescence spectra at different temperatures were regulated with a Thermostatic circulatorsr (HX-1005). Fluorescence lifetimes of samples were taken on a QuantaMater 8000 fluorescence spectrometer. Cell toxicity was determined using a microplate analyzer (Tecan Austria GmbH, Austria), and cell fluorescence images were taken using a focused laser scanning microscope (Olympus Fluoview FV3000).

Cytotoxicity assessment

MTT assay was used to evaluate the cytotoxicity of P1 on Hela cells**.** The

concentration of the cell suspension was adjusted to 10×10^4 cells /mL, and the Hela cell suspension (100 μL/ well) was added to the 96-well plate. After that, the cells were incubated for 24 h at 37°C in an incubator with 5% CO_2 content. After discarding the old medium, different concentrations of Z-2 samples were added to complete medium (0.6, 0.4, 0.2, 0.1, 0.05 mg/mL, respectively), and the culture was continued in the incubator for 24 h. 10 μL of MTT solution was added to each well, and the old cells were incubated with the new cells for 4 h. Finally, the medium containing MTT was removed, and 150 μL DMSO was added to each well. Then, DMSO was shaken at a low speed (100 rpm) for 10 min on a decolorization shaker, and the absorption value was measured at 492 nm wavelength with a microplate reader.

Cell imaging

HeLa cells were seeded in 6-well plates at 300,000 cells per well and incubated with 5% FBS and 1% penicillin/streptomycin (PS) for another 24 h at 37°C and 5% CO_2. The polymer P1 solution at a concentration of 0.5mg/ml was added to the Wells, and the cells were incubated for another 1h, washed with PBS buffer 2-3 times, and fixed with 4% cell fixative. Finally, HeLa cell imaging was performed with a laser confocal microscope under different imaging channels.

Synthesis

P1 was prepared by a simple one-pot epoxy ring-opening polymerization method. 1,3-dihydroxyacetone (0.9 g, 0.01 mol), Diglycidyl 1,2-cyclohexanedicarboxylate (2.843 g, 0.01 mol) and tetrabutylammonium bromide (0.161 g, 0.0005 mol) were placed in a three-necked flask and stirred at 115 °C for 24 h under N_2 protection. After cooling to room temperature, the mixture was dissolved in 30 mL of ethanol and then transferred to a dialysis bag with a cut-off molecular weight (MWCO) of 500 Da and dialyzed in a mixture of ultrapure water and ethanol with stirring for 72 hours. The solution containing the product was dried in a vacuum oven at 80°C for 24 h. The resulting Z was a dark brown viscous glue with a yield of 69%.

The synthetic procedure P 2is similar to that of P1 described above was obtained in 71% yield.

Molecular dynamics

All molecular dynamics (MD) simulations were performed using the GROMACS 2020.6 suite of programs [1]. The force field parameters of P1 and P2 were built from the general amber force field (GAFF) [2]. The top file of small molecule is generated by Sobtop software [3]. All simulations are performed under periodic boundary conditions. we performed 5 ns MD simulation in 10 nm ×10 nm ×10 nm water box.

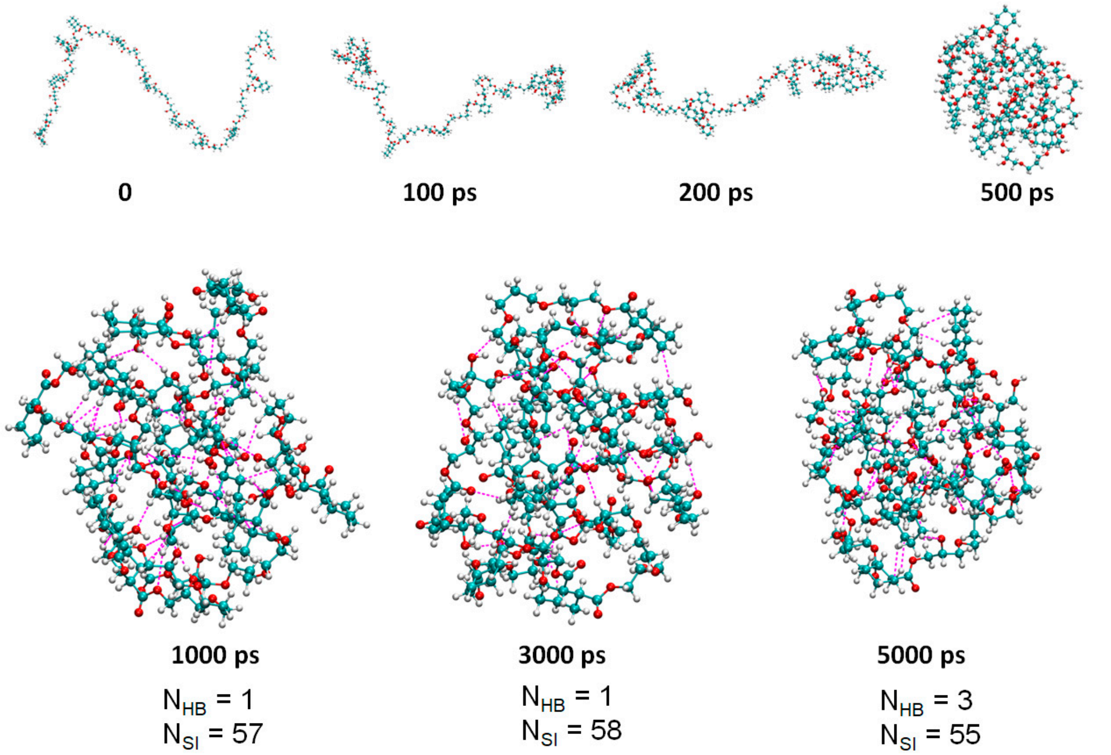

Figure S1. The aggregation state of P2 in 10 nm × 10 nm ×10 nm cubic water box at different times, and the number of hydrogen bonds (N_{HB}) and short interactions (N_{SI}) of the molecules at different times, the magenta dashed lines represent partial hydrogen bonds and short interactions.

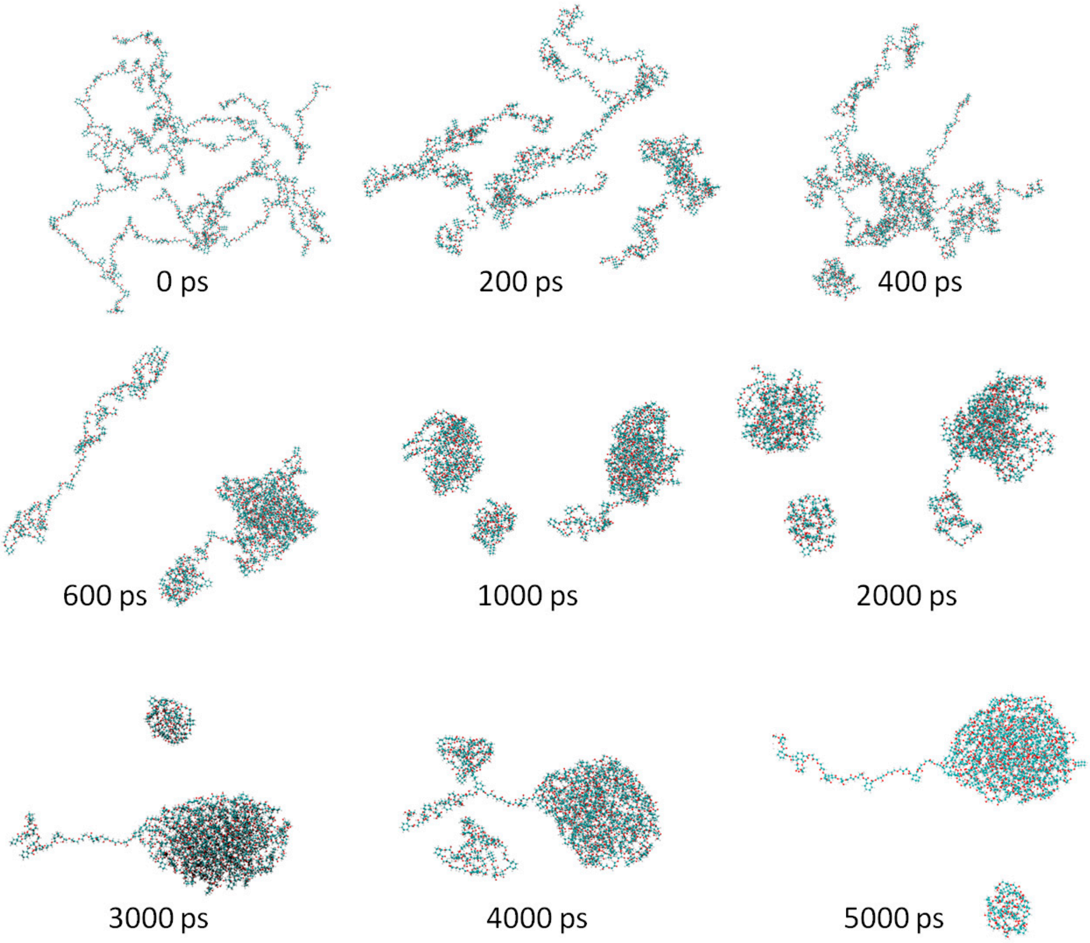

Figure S2. Molecular dynamics simulations of the aggregated state of a P1 polymer with polymer number 10 and repeating unit 11 in solution at different times.

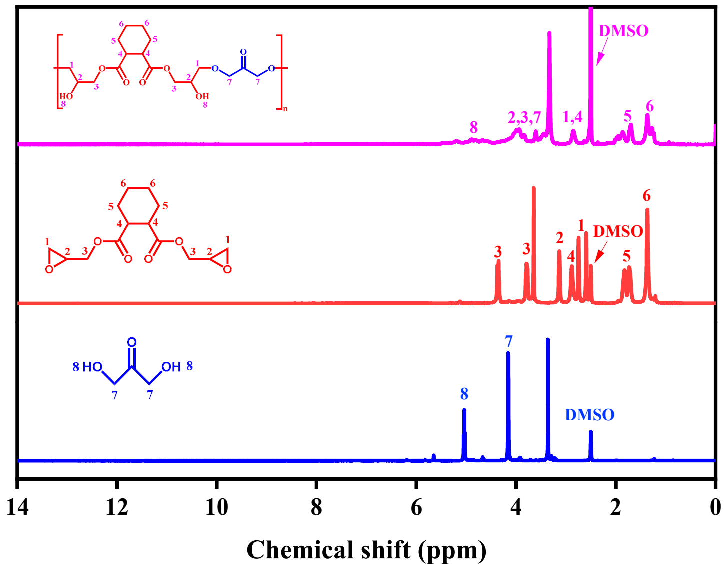

Figure S3. ^1H NMR spectrum of P1 and the corresponding synthetic raw materials.

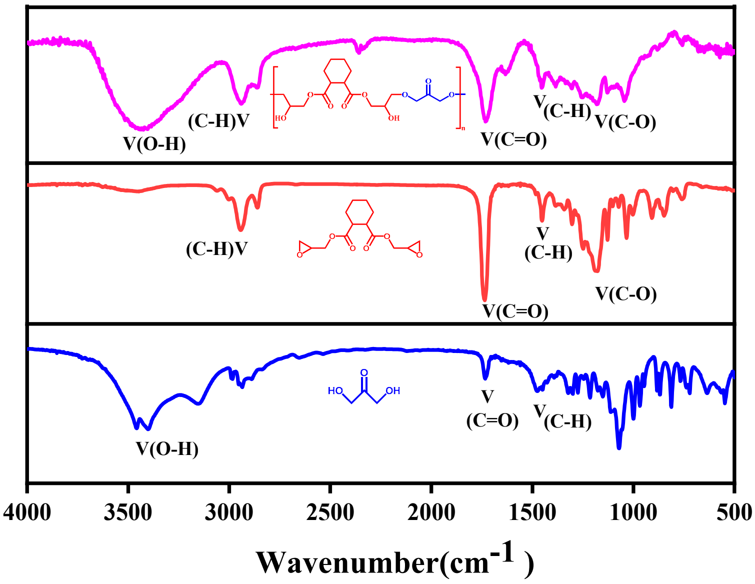

Figure S4. FT-IR spectrum of P1 and the corresponding synthetic raw materials.

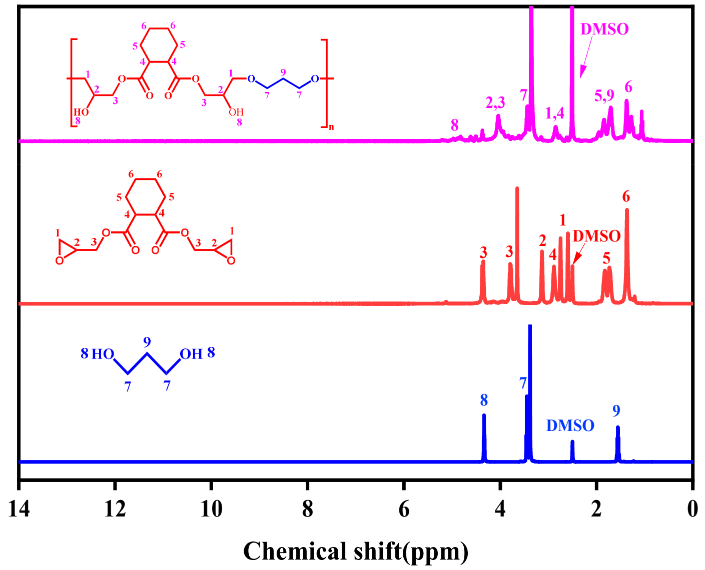

Figure S5. ^{1}H-NMR spectrum of P2 and the corresponding synthetic raw materials.

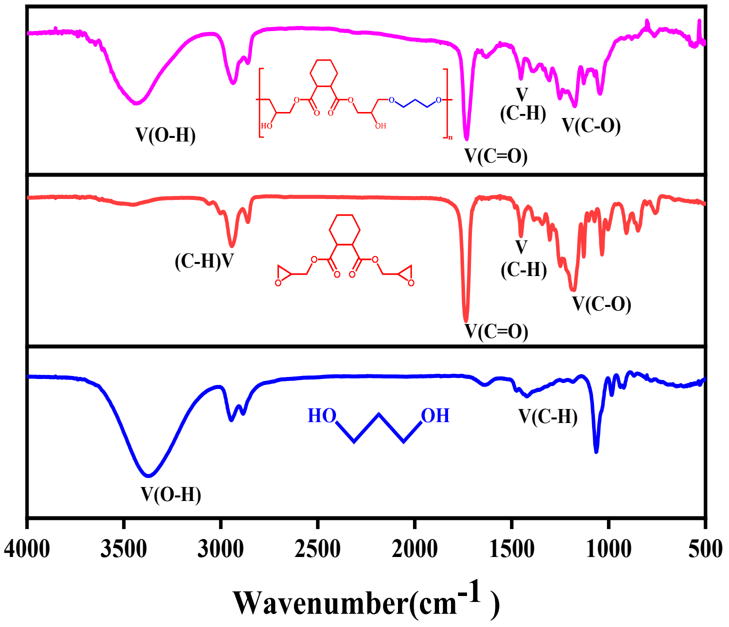

Figure S6. FT-IR spectrum of P2 and the corresponding synthetic raw materials.

Table S1 Molecular weight characterization of P1 and P2

Sample	Mn (g/mol)	Mw (g/mol)	Mz (g/mol)	PDI
P1	1715	3755	6341	2.2
P2	1145	1984	3163	1.7

M_n: Number average molecular weight.

M_w: Weight average molecular weight.

M_Z: Z-average molecular weight.

M_{Z+1}: Z+1-average molecular weight.

References

[1] Lindahl; Erik; Abraham; Hess; Spoel, v.d. GROMACS 2020.6 Source code, https://manual.gromacs.org/2020.6/download.html. Available online: (accessed on 10 July 2021).

[2] Galindo-Murillo, R.; Robertson, J.C.; Zgarbová, M.; Šponer, J.; Otyepka, M.; Jurečka, P.; Cheatham, T.E., III. Assessing the Current State of Amber Force Field Modifications for DNA. J. Chem. Theory Comput. 2016, 12, 4114-4127.

[3] Lu, T. Sobtop：A tool of generating forcefield parameters and GROMACS topology file. Sobtop 1.0 (dev3.1), http://sobereva.com/soft/Sobtop, (accessed on 1, Aug, 2022).
